# Dynamic Electroosmotic Flows of Power-Law Fluids in Rectangular Microchannels

**DOI:** 10.3390/mi8020034

**Published:** 2017-01-24

**Authors:** Cunlu Zhao, Wenyao Zhang, Chun Yang

**Affiliations:** 1 Key Laboratory of Thermo-Fluid Science and Engineering of MOE, School of Energy and Power Engineering, Xi’an Jiaotong University, Xi’an 710049, China; wyalbertzhang@foxmail.com; 2 School of Mechanical and Aerospace Engineering, Nanyang Technological University, 50 Nanyang Avenue, Singapore 639798, Singapore; MCYang@ntu.edu.sg

**Keywords:** microfluidics, flow enhancement, electroosmosis, non-Newtonian fluids

## Abstract

Dynamic characteristics of electroosmosis of a typical non-Newtonian liquid in a rectangular microchannel are investigated by using numerical simulations. The non-Newtonian behavior of liquids is assumed to obey the famous power-law model and then the mathematical model is solved numerically by using the finite element method. The results indicate that the non-Newtonian effect produces some noticeable dynamic responses in electroosmotic flow. Under a direct current (DC) driving electric field, it is found that the fluid responds more inertly to an external electric field and the steady-state velocity profile becomes more plug-like as the flow behavior index decreases. Under an alternating current (AC) driving electric field, the fluid is observed to experience more significant acceleration and the amplitude of oscillating velocity becomes larger as the fluid behavior index decreases. Furthermore, our investigation also shows that electroosmotic flow of power-law fluids under an AC/DC combined driving field is enhanced as compared with that under a pure DC electric field. These dynamic predictions are of practical use for the design of electroosmotically-driven microfluidic devices that analyze and process non-Newtonian fluids such as biofluids and polymeric solutions.

## 1. Introduction

Nowadays, microfluidic devices find promising applications in a variety of fields, including chemical analysis, medical diagnostics and material synthesis etc. The ultimate goal of microfluidics is to replace conventional large-scale laboratories with single, disposable microchips. This leads to some distinctive advantages, such as fast analyses, low sample consumption and cost as well as minimum personnel requirements etc. Usually, a microfluidic device has to perform multiple types of liquid sample manipulation to finish one single analysis, for example, pumping, mixing, injection, dispensing, just to name a few, among which pumping is the most fundamental one [1,2,3]. In order to achieve an optimal design and a better control of microfluidic devices, one needs to have a fundamental understanding of the liquid pumping in microchannels. In general, two popular ways to pump liquids in microfluidic devices are pressure-driven flow and electroosmotic flow. The problem for pressure driven flow is that it becomes increasingly difficult to pump liquids as channel size reduces to micron and submicron range. Electroosmotic flow however does not suffer from this problem, thereby providing an efficient way of pumping in microfluidic devices. In addition, electroosmotic flow possesses other advantages over pressure-drive flow, such as ease of fabrication and control, no need for moving parts, and easy integration with electronic controlling circuits for automation etc. Particularly, the plug-like velocity profile in electroosmotic flow minimizes the sample dispersion, which is essential for high-resolution separation in capillary electrophoresis [4].

Viscous relaxation or diffusion is the characteristic time scale for microscale liquid flow to develop to steady-state, and typically it is in the order of milliseconds [5]. An increasing number of practical microfluidic applications involving electroosmotic flows are in the sub-millisecond range, such as high-speed electrophoretic separation [6,7,8], the decoupling of particle velocity and background electroosmotic flow velocity with pulsed electric fields [9] as well as microfluidic pumping and mixing with alternating current (AC) or modulated direct current (DC) fields [10,11,12]. Therefore, understanding the dynamic characteristics of electroosmotic flows is highly important for these microfluidic applications. Previous theoretical studies of dynamic characteristics of electroosmosis have been focusing on Newtonian fluids under various modes of suddenly applied external fields [9,13,14,15,16,17,18,19,20]. In addition, experimental investigation of the dynamics of electroosmotic flows of Newtonian liquids was performed by utilizing state-of-the-art micro-particle image velocimetry (micro-PIV) techniques [21].

However, microfluidic devices are practically used to process biofluids (such as solutions of blood and DNA) which cannot be treated as Newtonian fluids and are usually characterized with viscosities dependent on the rate of shear. Therefore, the more general Cauchy momentum equation with a proper constitutive law, instead of the Navier–Stokes equation, should be used to describe flow characteristics of such fluids. Among various constitutive laws for non-Newtonian fluids, power-law constitutive law is the simplest yet most popular one. It has been shown to be suitable for the description of pressure-driven flows of various non-Newtonian fluids, such as polymeric solutions [22,23] and blood solutions [24,25,26]. A number of recent investigations [27,28,29,30,31,32,33,34,35,36,37] already showed that electroosmotically driven flows of non-Newtonian fluids behave differently from those of Newtonian fluids. However, their attention was unanimously focused on the steady-state characteristics and the dynamic aspects were missing from these investigations.

At present, the dynamic characteristics of electroosmosis of non-Newtonian fluids were mainly investigated for viscoelastic fluids. The existing studies have analyzed the dynamic electroosmosis of viscoelastic fluids in slit channel [38,39], circular channel [40,41], semi-circular channel [42] and rectangular channel [43]. It was revealed that the presence of the viscoelasticity can essentially affect dynamic aspects of electroosmosis. For power-law fluids, the study is however quite rare. The most relevant work at the moment is by Deng et al. [44] who analyzed the unsteady electroosmotic flow in a rectangular microchannel. Yet, their investigation is limited to low channel zeta potential and a pure DC driving electric field. In the present study, we report numerical analyses of transient electroosmotic flows of power-law fluids in a rectangular microchannel driven by three modes of electric field, namely a pure DC electric field, a pure AC electric field and a combination of AC and DC electric field. Besides, our analyses are valid for arbitrary channel zeta potential. The numerical simulations are carried out by using the finite element method which is verified through a comparison with the exact solution available for Newtonian fluids. Parametric studies are performed to examine the effects of fluid rheology (fluid behavior index) on the dynamics of electroosmosis of power-law fluids.

## 2. Problem Formulation

**Figure 1:**
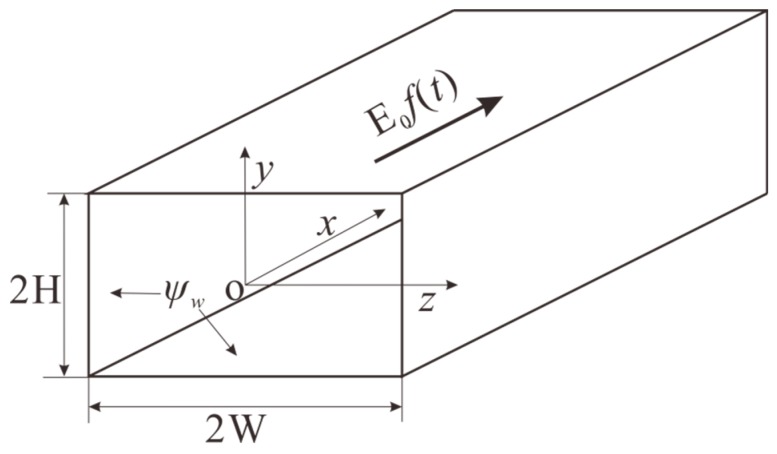
Electroosmotic flow system in a rectangular microchannel. The width of the channel is 2*W* and the depth of the channel is 2*H*. All the walls are uniformly charged with a zeta potential ψ*_w_*, and the dynamic electric field *E*_0_*f*(*t*) is applied along the axial direction of the microchannel. The zeta potential on the walls induces a near-wall electric double layer (EDL) which has a non-zero charge density. Then, interaction of the external electric field with the non-zero charge density induces a driving force for electroosmosis.

Figure 1 shows the dimensions of the microchannel and the coordinate system adopted in the present work. The channel is filled with a liquid solution having a dielectric constant of ε*_r_*. It is assumed that all channel walls are uniformly charged with a zeta potential of ψ*_w_*, and the liquid solution exhibits a typical non-Newtonian behavior which is described by the well-known power-law model. As soon as an external dynamic electric field *E*_0_*f*(*t*) is imposed along the *x*-axis direction, the fluid in the microchannel is set in motion due to electroosmosis. *f*(*t*) is a time-dependent function characterizing the dynamic behavior of the applied electric field. In this study, we consider three different modes of electric fields: a DC driving electric field with *f*(*t*) = 1, an AC driving electric field with *f*(*t*) = sin(ω*t*) and a combination of AC and DC electric fields with *f*(t) = 1 + εsin(ω*t*), in which ω and ε are the frequency and the amplitude of the AC component in the combined electric field. Because of the geometrical symmetry, the analysis would be restricted in the first quadrant of *z*-*y* plane.

### 2.1. Electric Field in the EDL

As aqueous solution in the microchannel contacts the charged wall, a thin charged solution layer forms near the wall to neutralize the surface charge on the channel wall. This layer is commonly referred to as the electric double layer (EDL). According to the electrostatic theory, electric potential distribution in the EDL region is governed by Poisson equation which can be expressed as

(1) $$\frac{\partial^{2}\psi }{\partial y^{2}}+\frac{\partial^{2}\psi }{\partial z^{2}}=-\frac{\rho_{e}}{\varepsilon_{0}\varepsilon_{r}}$$

where ε_0_ is the electric permittivity of vacuum, ρ*_e_* is the net charge density in the *EDL* region, and can be related to the EDL potential via (by invoking assumptions of Boltzmann distribution and *z_v_*:*z_v_* symmetric electrolyte) [45]

(2) $$\rho_{e}=\left(n_{+}-n_{-}\right)z_{v}e=-2z_{v}en_{\infty }\sinh\left(\frac{z_{v}e\psi }{k_{B}T}\right)$$

where *n*_+_ and *n*_−_ are respectively number of concentrations of cations and anions in the EDL region. *n*_∞_ and *z_v_* are the bulk number concentration and the valence of ions, respectively. *e* is the elementary charge, *k_B_* is the Boltzmann constant, and *T* is the absolute temperature.

Introducing dimensionless groups: $\bar{y}$ = *y/D_h_*, $\bar{z}$ = *z/D_h_*, *K* = *κD_h_*, and $\bar{\psi }$ = *z_v_e*ψ/(*k_B_T*), then substituting Equation (2) into Equation (1), one can show that electrical potential profile in the EDL is governed by the so-called Poisson–Boltzmann equation

(3) $$\frac{\partial^{2}\bar{\psi }}{\partial {\bar{y}}^{2}}+\frac{\partial^{2}\bar{\psi }}{\partial {\bar{z}}^{2}}=K^{2}\sinh\bar{\psi }$$

which is subject to the following boundary conditions

(4) $${\bar{\psi }|}_{\bar{y}=H/D_{h}}={\bar{\psi }}_{w},\;{\bar{\psi }|}_{\bar{z}=W/D_{h}}={\bar{\psi }}_{w}$$

(5) $${\frac{\partial \bar{\psi }}{\partial \bar{y}}|}_{\bar{y}=0}=0,\;{\frac{\partial \bar{\psi }}{\partial \bar{z}}|}_{\bar{z}=0}=0$$

In the above equations, *D_h_* represents the hydrodynamic diameter of the rectangular microchannel and is defined as *D_h_* = 4*HW*/(*H + W*), the dimensionless wall zeta potential is given by ${\bar{\psi }}_{w}=z_{v}e\psi_{w}/\left(k_{B}T\right)$, and the Debye length κ^−1^ is defined as κ^−1^ = [ε_0_ε*_r_k_B_T/*(2*e*^2^$z_{v}^{2}$*n*_∞_)]^1/2^.

### 2.2. Electroosmotic Flow of Power-Law Fluids

When an external electric field is applied, the flow of an incompressible power-law liquid induced by electroosmosis is jointly governed by the general Cauchy momentum equation and the continuity equation, i.e.,

(6) $$\rho \left[\frac{\partial V}{\partial t}+\left(V\cdot \nabla \right)V\right]=-\nabla p+\nabla \cdot \left[2\mu \left(\Gamma \right)\Gamma \right]+F$$

(7) ∇⋅V=0

where **V** is the velocity vector, ρ is the density of the liquid, *p* is the pressure, **F** is the body force vector, **Γ** is the rate of strain tensor and is given by **Γ** = [∇**V** + (∇**V**)*^T^*]/2. μ(**Γ**) is the dynamic viscosity and generally is a function of the magnitude of **Γ** tensor, Γ. The present work considers a power-law non-Newtonian fluid, and its dynamic viscosity is given by

(8) $$\mu \left(\Gamma \right)=m{\left(2\Gamma \right)}^{n-1}$$

where *m* is the flow consistency index, and *n* is the flow behavior index. Shear-thinning (also termed as pseudoplastic) behavior is defined by *n* < 1, and it indicates that the fluid viscosity decreases with the increasing rate of shear. The pseudoplastic effect commonly exists in polymeric fluids which are subject to the high rate of shear, as is developed in microchannels and nanochannels. Newtonian behavior is defined by *n* = 1. Shear-thickening (also termed as dilatant) behavior is defined by *n* > 1, and it shows that the fluid viscosity increases with the increasing rate of shear. The dilatant effect is unusual and rarely encountered in practical applications.

For the unidirectional electroosmotic flow considered here, the velocity vector can be simplified as

**V** = *u*(*y*,z,*t*) **e***_x_* (9)

where *u* is the *x*-component of velocity and **e***_x_* the is unit vector along the *x*-direction. Clearly, the continuity Equation (7) is automatically satisfied for the velocity field given by Equation (9). Furthermore, for electroosmotic flow, the only driving force is due to the interaction of the applied electrical field *E*_0_*f*(*t*) with the net charge density ρ*_e_* in the EDL region. In the present system shown in Figure 1, such body force acts only along the *x* direction, and is expressed as

(10) $$F=\rho_{e}E_{0}f\left(t\right)e_{x}$$

For an open-end, horizontally placed channel, there is no induced pressure gradient along the channel and hence the pressure gradient term in the Cauchy momentum equation can be neglected.

Besides the nondimensional groups used in the previous subsection, we introduce additional nondimensional parameters

(11) $$\bar{t}=\frac{\mu_{0}}{\rho D_{h}^{2}}t,\;\bar{u}=\frac{u}{u_{0}},\;\bar{m}=\frac{m{\left(2n_{\infty }k_{B}T\right)}^{n-1}}{\mu_{0}^{n}},\;{\bar{E}}_{0}=\frac{z_{v}eD_{h}E_{0}}{k_{B}T}$$

and take into account the aforementioned simplifications, then the nondimensional version of Equation (6) reads

(12) $$\frac{K^{2\left(n-1\right)}}{\bar{m}}\frac{\partial \bar{u}}{\partial \bar{t}}=\frac{\partial }{\partial \bar{y}}\left[\bar{\mu }\left(\bar{\Gamma }\right)\frac{\partial \bar{u}}{\partial \bar{y}}\right]+\frac{\partial }{\partial \bar{z}}\left[\bar{\mu }\left(\bar{\Gamma }\right)\frac{\partial \bar{u}}{\partial \bar{z}}\right]-\frac{K^{2n}}{\bar{m}}{\bar{E}}_{0}\bar{f}\left(\bar{t}\right)\sinh\left(\bar{\psi }\right)$$

where $\bar{\mu }\left(\bar{\Gamma }\right)$ can be formulated as

(13) $$\bar{\mu }\left(\bar{\Gamma }\right)={\left(2\bar{\Gamma }\right)}^{n-1}={\left[{\left(\frac{\partial \bar{u}}{\partial \bar{y}}\right)}^{2}+{\left(\frac{\partial \bar{u}}{\partial \bar{z}}\right)}^{2}\right]}^{\frac{n-1}{2}}$$

In Equation (11), μ_0_ denotes the viscosity of Newtonian fluids and it has the same magnitude as the flow consistency index *m*. *u*_0_ then can be viewed as the Helmholtz–Smoluchowski velocity for Newtonian liquids over a solid surface with zeta potential being equal to the thermal voltage (*k*_B_*T*/(*z_v_e*)) under an electric field strength of *k*_B_*T*/(*z_v_eD_h_*), and is given by

(14) $$u_{0}=\frac{\varepsilon_{0}\varepsilon_{r}}{\mu_{0}}\frac{k_{B}T}{z_{v}e}\frac{k_{B}T}{D_{h}z_{v}e}$$

It is worth mentioning that reference quantities for time and velocity are independent of the rheological properties of fluids (*n* and *m*). Choosing reference quantities in such a manner is convenient when discussing the effect of fluid rheology on magnitudes of both electroosmotic velocity and transient start-up time in Section 4.

The initial and boundary conditions applicable to Equation (12) are

(15) $${\bar{u}|}_{\bar{t}=0}=0$$

(16) $${\frac{\partial \bar{u}}{\partial \bar{y}}|}_{\bar{y}=0}=0,\;{\frac{\partial \bar{u}}{\partial \bar{z}}|}_{\bar{z}=0}=0$$

(17) $${\bar{u}|}_{\bar{y}=H/D_{h}}=0,\;{\bar{u}|}_{\bar{z}=W/D_{h}}=0$$

## 3. Numerical Method and Model Validation

In the present analysis, both EDL potential field and electroosmotic flow field are solved in the partial differential equation (PDE) module of finite element numerical analysis package COMSOL Multiphysics 5.1 (COMSOL, Inc., Stockholm, Sweden). In the PDE module, the general form of PDE is given in terms of a series of coefficients and a source term which are left for the users to specify for formulating their models. These coefficients and the source term can be either constants or variables, thereby generating high flexibility for handling PDEs. In our work, a PDE governing the EDL potential (Equation (3)) and a PDE governing electroosmotic flow field (Equation (12)) are both constructed from the general form of PDE in Comsol. Through the source term $\sinh\left(\bar{\psi }\right)$ in Equation (12), these two PDEs are coupled together.

In order to check the validity of the present model, we compared our numerical result with the exact result [46] derived for the starting electroosmotic flow of Newtonian fluids in a rectangular microchannel. However, their result was obtained under the Debye–Hückel linear approximation which assumes a small zeta potential on the channel wall. Thus, in the numerical validation, a small zeta potential (ψ*_w_ = −k_B_T/*(*z_v_e*) ≈ −25 mV for monovalent electrolytes at room temperature) was prescribed for the channel walls and geometric dimensions of the microchannel were chosen as *2H* = 10 μm and *2W* = 15 μm. The working fluid flowing in the microchannel is the Newtonian solution (a special power-law fluid with flow behavior index *n* = 1) of a symmetric electrolyte (*z_v_*:*z_v_*), say NaCl. The bulk ionic number concentration was set to *n_∞_* = 6.022 × 10^20^/m^3^. The dielectric constant of electrolytic solution was taken to be the same as that of room-temperature water, namely ε*_r_* = 78.5. In electroosmotic flows, the velocity experiences steep changes in the EDL region near the channel walls. Therefore, in the present analysis, the mesh near the channel wall is finest to ensure that the velocity change in the EDL can be captured, and at least ten cells are positioned inside the EDL region. The mesh size increases towards the center region of the cross-section with mesh ratios of 1.04 and 1.03 in *y* and *z* directions, respectively. The maximal cell has dimensions of Δ*y*/*D_h_* = Δ*z*/*D_h_* = 1.72 × 10^−2^. The time step used in this study is controlled to satisfy Δ*t*/*T* ≤ 2 × 10^−3^, where *T* is the start-up time for DC-driven electroosmosis or the period of the AC electric field. The calculated solutions were carefully validated to exclude both mesh dependency and time-step dependency. Mesh-independence was examined for two different mesh systems whose total cell numbers are 15,000 (150 × 100) and 60,000 (300 × 200), respectively. Two different time steps, i.e., 1 × 10^−3^ and 5 × 10^−4^, were also examined. It was found that calculated flow rate differences under two examinations were both less than 1%. Therefore, mesh independence and time-step independence were confirmed, and then the mesh system with 15,000 cells (150 × 100) and a time step of 1 × 10^−3^ were applied in the study. The UMFPACK solver was used to solve the system with relative tolerances of spatial and temporal solutions both being 10^−6^.

**Figure 2:**
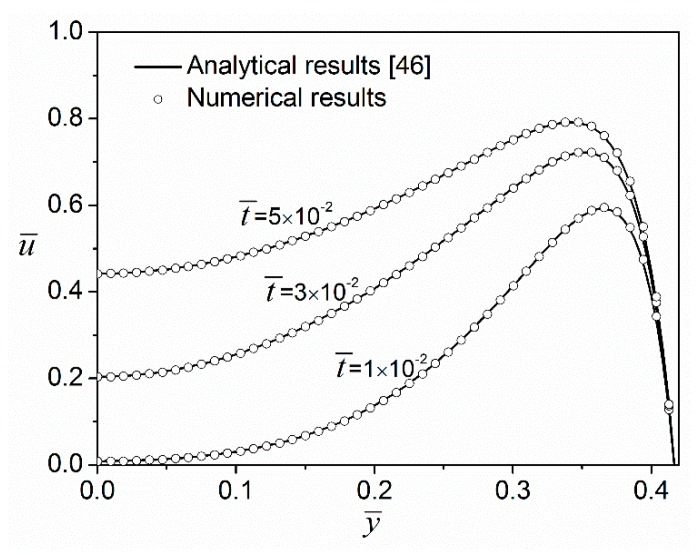
Transient velocity profiles along the $\bar{y}$ axis when $\bar{z}=0$ at three different time instants for Newtonian fluids (*n* = 1.0) under a DC electric field ${\bar{E}}_{0}=1$ and a zeta potential ${\bar{\psi }}_{w}=-1$.

Figure 2 shows the velocity profiles at $\bar{z}=0$ for three different time instants computed with the analytical formula [46] and our Comsol model. It can be seen from this plot that the numerical results of velocity distributions obtained from the Comsol model at three different time instants agree perfectly well with those obtained from the existing analytical model, which validates the high robustness and accuracy of the Comsol model.

## 4. Results and Discussion

To predict dynamic behaviors of electroosmotic flows of power-law fluids under various modes of electric fields, we take values of some parameters to be the same as those in Section 3, i.e., 2*H* = 10 μm, 2*W* = 15 μm, *n_∞_* = 6.022 × 10^20^ m^−3^. The dynamic viscosity of Newtonian fluids is set to be μ_0_ = 9 × 10^−4^ Pa·s (the same as room-temperature water) and flow consistency index of power-law fluids is taken as *m* = 9 × 10^−4^ Pa·s*^n^* (the same magnitude as dynamic viscosity of Newtonian fluids). The corresponding dimensionless electrokinetic parameter *K* = 40, which makes sure that the microchannel has a moderately thin EDL, and thus the dynamic momentum transfer from the EDL to the bulk flow can be identified.

### 4.1. Transient Electroosmotic Flows of Power-Law Fluids under DC Electric Fields

**Figure 3:**
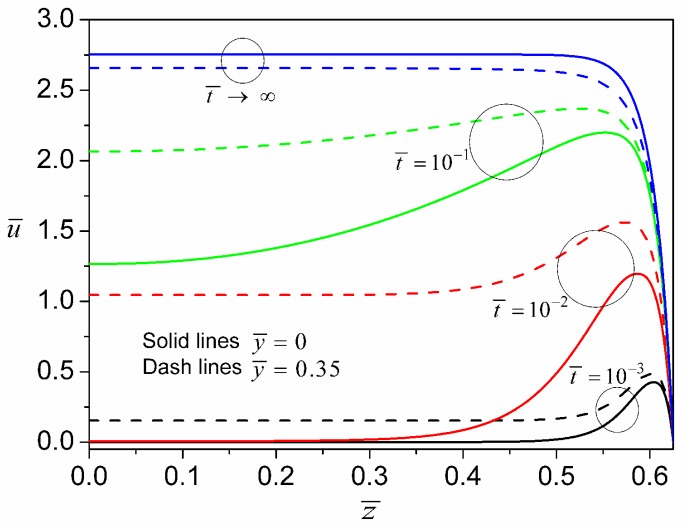
Transient evolution of axial velocity profiles for a power-law fluid with *n* = 0.8 and ${\bar{\psi }}_{w}=-1$ due to an applied DC electric field ${\bar{E}}_{0}=1$.

Figure 3 shows electroosmotic velocity profiles of a power-law fluid with *n* = 0.8 at different time instants. Initially, the liquid in the whole microchannel is quiescent (not shown in Figure 3). As soon as the electric field is applied, the liquid within the EDL starts to flow immediately, but the bulk liquid in the middle portion of microchannel remains stationary. As the dimensionless time evolves to 10^−3^, at $\bar{y}=0$, the velocity reaches a local maximum near the vertical side wall (inside the EDL of side wall), and then drops gradually to zero as the distance is away from the side wall. At $\bar{y}=0.35$ (very near the top wall), there is similarly a local maximal velocity near the side wall. However, the liquid far from the vertical side wall in this case is already in motion because of the EDL of the top wall. Moreover, the maximal velocity at $\bar{y}=0.35$ is higher than that at $\bar{y}=0$. This is because at $\bar{y}=0.35$ near the vertical side wall (around the right upper corner of channel cross-section), the liquid is actuated by the electrostatic body force due to EDLs on both the top and side walls; while at $\bar{y}=0$ near the vertical side wall, the liquid is actuated by the electrostatic body force due to the EDL on the side wall alone. In electroosmotic flows, the driving force is only present in the EDL region, and the generation of momentum is then also limited in the EDL region. As time evolves, the fluid velocity within the EDL continues to increase; at the same time, the bulk fluid starts moving due to the gradual transfer of momentum from the EDL to bulk liquid (see velocity profiles at $\bar{t}=10^{-2}$ and $\bar{t}=10^{-1}$). When the flow develops to the steady state ($\bar{t}\to \infty$), the velocity distribution exhibits a plug-like profile. It is also observed that the velocity profile at $\bar{y}=0.35$ develops faster than that at $\bar{y}=0$, which is peculiar to rectangular channels. This effect can be ascribed to the fact that at $\bar{y}=0.35$ the driving force is present along the entire $\bar{z}$ axis, while at $\bar{y}=0$ the driving force is present only near the vertical side wall.

**Figure 4:**
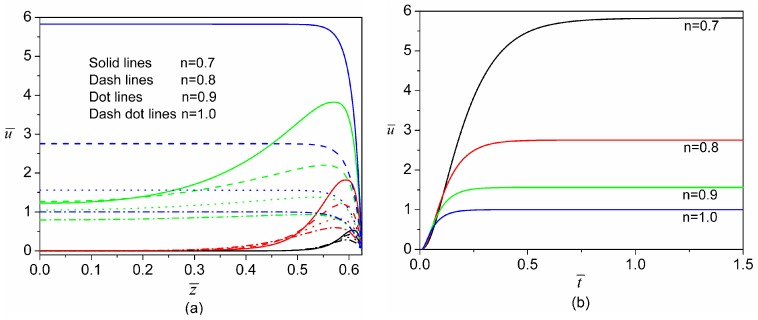
Comparison of transient development of electroosmosis for four different fluid behavior indices (*n* = 0.7, 0.8, 0.9 and 1.0) when ${\bar{E}}_{0}=1$ and ${\bar{\psi }}_{w}=-1$. (**a**) Transient velocity profiles along $\bar{z}$ axis at $\bar{y}=0$. There are four groups of velocity profiles in this plot and each group represents the velocity profiles of four flow behavior indices at a specific time instant: the group with black color is at $\bar{t}=10^{-3}$, the group with red color is at $\bar{t}=10^{-2}$, the group with green color is at $\bar{t}=10^{-1}$ and the group with blue color is at the steady state ($\bar{t}\to \infty$); (**b**) Time evolution of velocity at the center of cross-section ($\bar{z}=\bar{y}=0$).

Usually, the fluid behavior index (*n*) is varied by the addition of polymers into the solutions which also changes the value of zeta potential. This indicates that the zeta potential is practically a function of *n*. However, at present, the quantitative relation between zeta potential and *n* is unclear and remains to be investigated. Therefore, for convenience, the zeta potential is assumed to be an independent variable which is not influenced by *n* in the current study. Such an assumption is widely adopted in the literature for study of electroosmotic flow of non-Newtonian fluids [27,28,29,30,31,32,33,34,35,36,37]. Figure 4 characterizes the transient development of electroosmosis of power-law fluids with different fluid behavior indices. The velocity in the whole channel domain is zero at *t* = 0 for all values of the fluid behavior index (not shown in Figure 4). As shown in Figure 4a, when dimensionless time evolves to 10^−3^, the fluids with smaller fluid behavior indices acquire higher velocities inside the EDL region near the channel wall. The velocity inside the EDL becomes higher as time evolves, and at the same time the momentum generated inside the EDL gradually diffuses to the bulk. At $\bar{t}=10^{-2}$, the fluids with smaller fluid behavior indices still have higher velocities inside the EDL region, while outside the EDL the velocity for a larger fluid behavior index surpasses that for a smaller fluid behavior index. This feature indicates that the momentum transfer is faster for a larger fluid behavior index due to the stronger viscous coupling between the EDL and the bulk liquid. At the steady state ($\bar{t}\to \infty$), normalized velocities in the bulk flow for four fluid behavior indices all increase to their corresponding constant values, which are typical for electroosmotically-driven flows. Furthermore, at the steady state, the velocity profiles for smaller fluid behavior indices become more plug-like and also the magnitude of bulk velocity is larger for a smaller fluid behavior index. As is the case for *n* = 0.7, the steady sate bulk liquid velocity is more than five times higher than that of Newtonian fluids (*n* = 1), which implies that the electroosmotic pumping of shearing-thinning fluids is far more efficient than that of Newtonian fluids. For situations where we have large-sized channels or thin EDLs (i.e.,K≫1), it can be expected that the power-law fluid in the entire microchannel moves with a uniform bulk velocity. Consequently, the constant bulk velocities for various values of flow behavior index can be effectively seen as the so-called Helmholtz–Smoluchowski velocities in electrokinetics of power-law fluids (i.e., electrophoresis of particles in power-law fluids and electroosmosis of power-law fluids). In addition, it is shown in Figure 4b that the fluids with larger fluid behavior indices approach the steady state more quickly. This is because the fluids with larger fluid behavior indices are more viscous and then the momentum generated inside the EDL can be transferred more promptly to the center portion of the channel.

**Figure 5:**
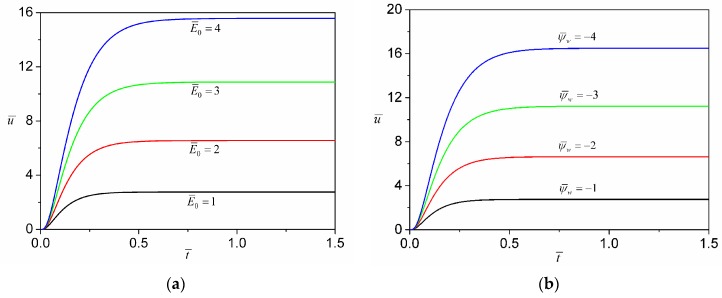
(**a**) Comparison of transient development of the axial velocity at the transverse center ($\bar{z}=\bar{y}=0$) under different magnitudes of external electric field strength when *n* = 0.8 and ${\bar{\psi }}_{w}=-1$; (**b**) Comparison of transient development of the axial velocity at the transverse center ($\bar{z}=\bar{y}=0$) for different magnitudes of zeta potential when *n* = 0.8 and ${\bar{E}}_{0}=1$.

The effects of DC field strength and wall zeta potential on the transient development of electroosmosis are shown in Figure 5. The transient start-up time during which the velocity develops from zero to the steady state becomes shorter when the strength of electric field/zeta potential is decreased, and the magnitude of steady-state velocity increases nonlinearly with the increase of the strength of external electric field/zeta potential. These characteristics clearly differ from electroosmotic flows of Newtonian fluids for which the transient start-up time is independent of the strength of electric field/zeta potential, and also the magnitude of steady-state velocity increases *linearly* with the increasing strength of electric field/zeta potential [17,46,47].

### 4.2. Transient Electroosmotic Flows of Power-Law Fluids under AC Electric Fields

**Figure 6:**
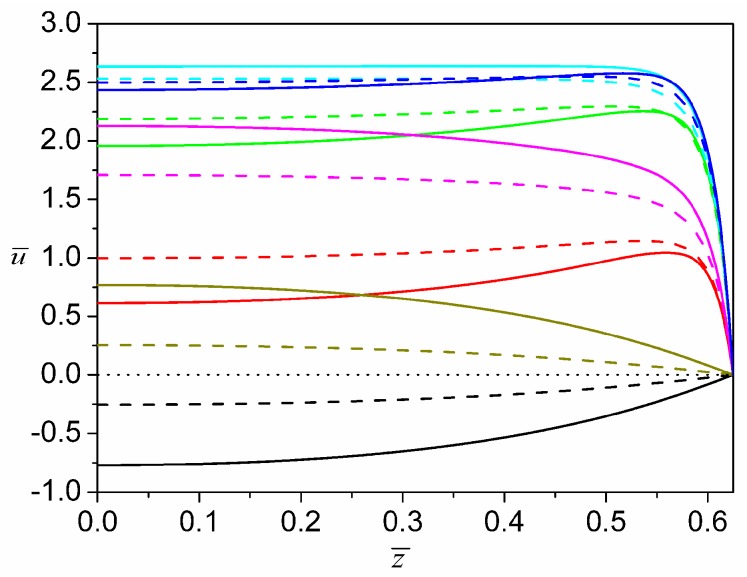
Steady-state oscillating axial velocity profiles of a power-law fluid with *n* = 0.8 at different phases in a half period from $\bar{\omega }\bar{t}=0$ to $\bar{\omega }\bar{t}=\pi$ when ${\bar{E}}_{0}=1$, ${\bar{\psi }}_{w}=-1$ and $\bar{\omega }=\pi$. There are seven groups of velocity profiles differentiated by different colors and each group represents the profiles at a given AC phase. In each group, the solid line is the velocity profile at $\bar{y}=0$ and the dash line is the profile at $\bar{y}=0.35$. The group with black color represents the profiles at $\bar{\omega }\bar{t}=0$; the group with red color represents the profiles at $\bar{\omega }\bar{t}=\pi /5$; the group with green color represents the profiles at $\bar{\omega }\bar{t}=2\pi /5$; the group with blue color represents the profiles at $\bar{\omega }\bar{t}=\pi /2$; the group with cyan color represents the profiles at $\bar{\omega }\bar{t}=3\pi /5$; the group with magenta color represents the profiles at $\bar{\omega }\bar{t}=4\pi /5$ and the group with dark-yellow color represents the profiles at $\bar{\omega }\bar{t}=\pi$. On the straight dot line, $\bar{u}=0$.

In this particular investigation, the electroosmotic flow is driven by a pure AC electric field. Then, in the simulation, we choose $\bar{f}\left(\bar{t}\right)=\sin\left(\bar{\omega }\bar{t}\right)$ and the corresponding dimensionless frequency to be $\bar{\omega }=\omega \rho D_{h}^{2}/\mu_{0}=\pi$. Figure 6 presents the steady-state development of the axial velocity profile in the transverse section for a half period (from phase $\bar{\omega }\bar{t}=0$ to phase $\bar{\omega }\bar{t}=\pi$) when ${\bar{E}}_{0}=1$ and ${\bar{\psi }}_{w}=-1$. At $\bar{\omega }\bar{t}=0$, although the electric field strength is zero, the flow field lags behind the electric field and the preceding negative electric field strength causes liquid in the microchannel to move along the negative *x* direction (negative velocity). As time elapses, the liquid within the EDL is rapidly driven to the positive *x* direction. Then, at the same time, the momentum transfer from the EDL to the bulk flow progresses, leading to the expansion of the positive-velocity region from the EDL towards the central region of the microchannel. Until phase $\bar{\omega }\bar{t}=\pi /5$, the positive-velocity region already expands to occupy the entire microchannel. From phase $\bar{\omega }\bar{t}=\pi /5$ to phase $\bar{\omega }\bar{t}=\pi /2$, the momentum transfer from the EDL to the bulk flow is enhanced by the increasing electric field strength, and thus the velocity in the whole channel domain continues to grow. After phase $\bar{\omega }\bar{t}=\pi /2$, the strength of the electric field begins to decrease, and the liquid the within EDL responds instantaneously to such change. Therefore, there is a slight reduction in the positive axial velocity near the walls. Nevertheless, the positive axial velocity in the microchannel center still increases due to the inertial acceleration (see profiles at phase $\bar{\omega }\bar{t}=3\pi /5$). After phase $\bar{\omega }\bar{t}=3\pi /5$ (such as phase $\bar{\omega }\bar{t}=4\pi /5$), the decrement of momentum inside the EDL expands towards the central region of channel, which makes the axial velocity in the bulk flow decrease. At phase $\bar{\omega }\bar{t}=\pi$, it is noted that the axial flow velocity profiles strongly resemble those at phase $\bar{\omega }\bar{t}=0$ in terms of their shapes. However, the direction of axial velocity is opposite to that at phase $\bar{\omega }\bar{t}=0$. During the second half period (from $\bar{\omega }\bar{t}=\pi$ to $\bar{\omega }\bar{t}=2\pi$), since the variation of an AC driving electric field is a mirror image of that during the preceding half period (from $\bar{\omega }\bar{t}=0$ to $\bar{\omega }\bar{t}=\pi$), it is quite understandable that the corresponding evolution of axial velocity profiles is also a mirror image (symmetric with respect to the dot line $\bar{u}=0$ in Figure 6) of the preceding half period.

**Figure 7:**
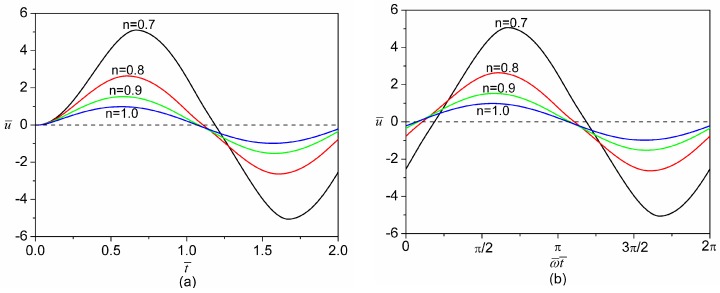

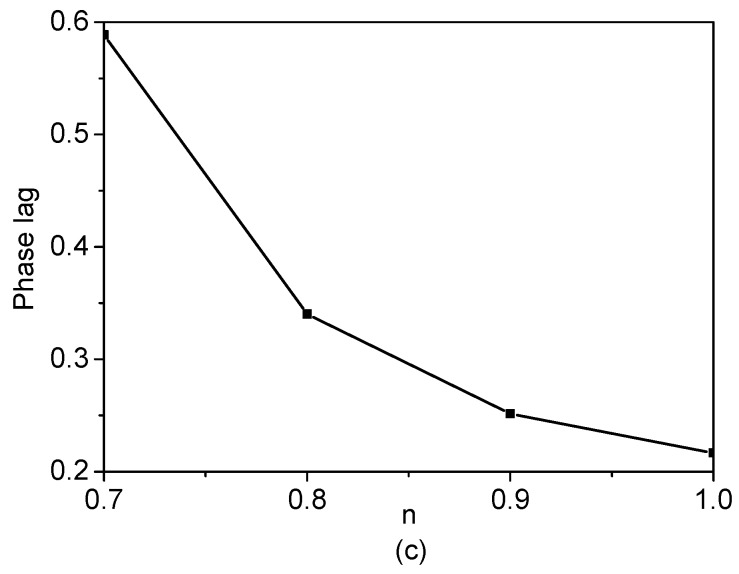
Comparison of the velocity evolution at the transverse center ($\bar{z}=\bar{y}=0$) of the microchannel for different values of flow behavior index (*n* = 0.7, 0.8, 0.9, 1.0) under an AC electric field with ${\bar{E}}_{0}$ = 1, $\bar{\omega }=\pi$ and ${\bar{\psi }}_{w}=-1$. (**a**) Start-up characteristics of the electroosmotic velocity; (**b**) Steady-state oscillation of the electroosmotic velocity; (**c**) Variation of the phase lag between velocity and AC field with the fluid behavior index at the steady-state oscillation.

Figure 7 presents the comparison of transient velocity development for different values of fluid behavior index at both the start-up stage and the steady-state oscillation. After turning on the AC electric field at $\bar{t}=0$, it is seen from Figure 7a that the fluid at the transverse center ($\bar{y}=\bar{z}=0$) remains quiescent for a very short period of time. At this moment, the momentum generated inside the EDL is still limited to the regions near the channel walls and therefore needs time to diffuse to the bulk liquid. After a certain amount of time, the momentum is gradually transferred to the bulk liquid and then the liquid starts to move. The fluid with a larger fluid behavior index responds more promptly to the applied AC field and then reaches the peak velocity more quickly. This is consistent with the case of the DC electric field in which the fluid with a larger fluid behavior index reaches the steady state more quickly. When the flow attains the steady-state oscillation (Figure 7b), the velocity generally lags behind the applied AC electric field, and the phase lag increases with the decrease of fluid behavior index, as is indicated in Figure 7c. We also note from Figure 7b that from one peak to its corresponding trough, power-law fluids with a smaller fluid behavior index experience more significant acceleration. Furthermore, the amplitude of oscillating velocity increases as the fluid behavior index decreases.

### 4.3. Enhancement of Electroosmotic Flows of Power-Law Fluids by AC/DC Combined Electric Fields

**Figure 8:**
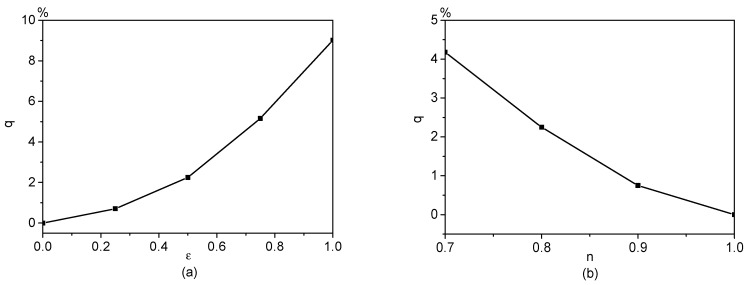
Flow enhancement of electroosmotic flows of power-law fluids by an AC/DC combined electric field with ${\bar{E}}_{0}=1$, $\bar{\omega }=\pi$ and ${\bar{\psi }}_{w}=-1$. (**a**) Variation of *q* with ε when *n* = 0.8; (**b**) Variation of *q* with the flow behavior index *n* when ε = 0.5.

For pressure-driven flows of power-law fluids, it is known that the flows can be enhanced by introducing one pulsatile pressure gradient to a constant pressure gradient [22,48]. Generally, this flow enchantment arises from the nonlinear relationship between the stress and the rate of strain which reduces effective viscosity of the liquids. Our investigation here proves that a similar concept can be used to enhance the electroosmotically-driven flow of power-law fluids by adding one AC electric field to a DC electric field. Particularly, the time characteristics of an AC/DC combined electric field is characterized by $\bar{f}\left(\bar{t}\right)=1+\varepsilon \sin\left(\bar{\omega }\bar{t}\right)$, where ε defines the amplitude of the AC component of the electric field. In addition, a percentile, *q* = 100% × (*Q*_ε_ − *Q*_0_)/*Q*_0_, is defined to quantify the flow enhancement due to the AC electric field. In the definition of this percentile, *Q*_ε_ is the flow rate due to an AC/DC combined electric field ${\bar{E}}_{0}\left[1+\varepsilon \sin\left(\bar{\omega }\bar{t}\right)\right]$, and *Q*_0_ is the flow rate due to a DC electric field ${\bar{E}}_{0}$ alone. The higher the *q* is, the more significant the flow enhancement is. Figure 8 shows the effects of AC amplitude and flow behavior index on *q*. It is clear that *q* increases with the increase of AC amplitude or the decrease of flow behavior index. These predictions are similar to the case of pressure-driven power-law fluid flow [22,48] in which the flow enhancement is amplified by increasing the amplitude of pulsatile pressure gradient or decreasing the flow behavior index.

## 5. Conclusions

We have presented a comprehensive numerical analysis of dynamic electroosmotic flows of power-law fluids in rectangular microchannels under three modes of electric fields. For the case of transient electroosmotic flow driven by a pure DC electric field, initially, the DC electric field drives the liquid within the EDL immediately to move in the axial direction. Then the momentum generated in the EDL gradually transfers to the bulk region of channel, which leads to a plug-like velocity profile at the steady state. Generally, the non-Newtonian nature of fluids complicates the transient dynamics of electroosmosis. It is observed that the flow with a higher fluid behavior index responds more promptly to the external DC electric field and reaches the steady state more quickly. Another prominent feature is that the transient start-up time becomes dependent on the strength of the electric field/zeta potential for power-law fluids.

For the case of a pure AC electric field, the results show that the flow in the microchannel initially shows a transient start-up after the immediate application of the electric field and finally attains a steady-state oscillation. The electroosmosis of fluid with a larger fluid behavior index demonstrates a faster response to the external AC electric field and consequently has a smaller phase lag behind the applied AC electric field. At last, for the case of an AC/DC combined electric field, it is shown that the flow is enhanced as compared to a pure DC electric field. This feature is similar to the flow enhancement in non-Newtonian fluid flows driven by a pulsatile pressure gradient. The results show that increasing the amplitude of AC field component or decreasing the flow behavior index can intensify the electroosmotic flow enhancement of power-law fluids. These conclusions are of practical significance because they can be of potential use in guiding the design of microfluidic analytical devices which involve electroosmotic flows of non-Newtonian fluids.
